# The unusual stoichiometry of ADP activation of the K_ATP_ channel

**DOI:** 10.3389/fphys.2014.00011

**Published:** 2014-01-28

**Authors:** Eric Hosy, Michel Vivaudou

**Affiliations:** ^1^Institut de Biologie Structurale, University Grenoble AlpesGrenoble, France; ^2^Laboratory of Excellence, Ion Channel Science and Therapeutics, CNRS, Institut de Biologie StructuraleGrenoble, France; ^3^CEA, DSV, Institut de Biologie StructuraleGrenoble, France

**Keywords:** K-ATP channel, sulfonylurea receptor, ABC transporter, ADP, hyperinsulinism

## Abstract

K_ATP_ channels, oligomers of 4 pore-forming Kir6.2 proteins and 4 sulfonylurea receptors (SUR), sense metabolism by monitoring both cytosolic ATP, which closes the channel by interacting with Kir6.2, and ADP, which opens it via SUR. SUR mutations that alter activation by ADP are a major cause of K_ATP_ channelopathies. We examined the mechanism of ADP activation by analysis of single-channel and macropatch recordings from *Xenopus* oocytes expressing various mixtures of wild-type SUR2A and an ADP-activation-defective mutant. Evaluation of the data by a binomial distribution model suggests that wild-type and mutant SURs freely co-assemble and that channel activation results from interaction of ADP with only 2 of 4 SURs. This finding explains the heterozygous nature of most K_ATP_ channelopathies linked to mutations altering ADP activation. It also suggests that the channel deviates from circular symmetry and could function as a dimer-of-dimers.

## Introduction

K_ATP_ channels are metabolic sensors that play key roles in cardioprotection and glycemia control. They are formed by association of a K^+^ channel subunit Kir6.x and a regulatory subunit, the sulfonylurea receptor SUR, of the ABC protein family (Moreau et al., 2005b; Nichols, 2006). The channel complex is a hetero-octamer with 4 Kir6.2 delimiting an ATP-inhibited inwardly rectifying K^+^ channel surrounded by 4 SUR subunits (Mikhailov et al., 2005). This stoichiometry, imposed by the presence of endoplasmic reticulum retention signals in Kir6.2 (Zerangue et al., 1999), appears independent of the nature of the SUR isoform, be it SUR1, SUR2, or a mixture of both (Chan et al., 2008; Wheeler et al., 2008). Intracellular ATP causes channel closure by binding to 1 of the 4 Kir6.2s (Markworth et al., 2000) while ADP, interacting with the nucleotide-binding domains (NBDs) of SUR in a Mg^2+^-dependent fashion, promotes its opening (Nichols, 2006). The conjunction of these effects underlies the ability of the channel to gate as a function of the cytoplasmic ATP/ADP ratio, and their deregulation is at the origin of several genetic diseases. In particular, SUR mutations that alter activation by ADP lead to recessive and dominant forms of pancreatic (Shyng et al., 1998; Huopio et al., 2000; Dunne et al., 2004) and cardiac disorders (Bienengraeber et al., 2004; Olson et al., 2007).

It is now recognized that nucleotidic regulations of the K_ATP_ channel involve intricate enzymatic mechanisms both within the channel subunits and in the environmental context of the cell (Zingman et al., 2002; Alekseev et al., 2005). Nonetheless, the relationships between activity of isolated channels and nucleotide concentrations remain consistent with classical ligand-receptor interactions. In particular, the relation between channel activation and ADP is well approximated by a non-cooperative bimolecular agonist-receptor model (Forestier and Vivaudou, 1993; Matsuo et al., 2002; Dupuis et al., 2008; Proks et al., 2010). Such a model provides a simple framework that accurately reflects the global response of the channel and bypasses detailed molecular intricacies. We used this model for the present study of the stoichiometry of ADP activation of the K_ATP_ channel using a SUR mutant that is ADP-activation defective.

## Materials and methods

Experimental conditions were as previously described by Hosy et al. (2007). Site-directed mutagenesis of mouse Kir6.2 and rat SUR2A (the kind gift of Dr. S. Seino, Chiba, Japan) was accomplished with the QuickChange kit (Stratagene). K_ATP_ channels were heterologously expressed in *Xenopus laevis* oocytes by microinjection of RNAs coding for the Kir6.2 and SUR2A subunits. Xenopus laevis oocytes were surgically removed from anesthetized Xenopus laevis females using procedures that conformed to European regulations for animal handling and experiments, and were approved by governmental services (Authorization N°38 08 10 granted to Michel Vivaudou by the local veterinary agency, Directeur Départemental des Services Vétérinaires, Ministère de l'Agriculture et de la Pêche, on 22 February 2008, valid until 06 July 2015) and the Institutional Ethical Committee (Ethical Committee of Commissariat à l'Energie Atomique et aux Energies Alternatives for animal experiments, assessment n°12-040 on 23 December 2012). The quality and concentration of *in-vitro* transcribed cRNAs were estimated by electrophoresis and spectrophotometry. *Xenopus laevis* oocytes were injected with 2 ng of Kir6.2 cRNA and 6 ng of various mixes of WT and mutated SUR2A cRNA. These amounts were reduced 100-fold for recordings of single channels. Before patch-clamp experiments, microinjected oocytes were incubated for more than 2 days at 19°C in Barth's solution (in mM: 1 KCl, 0.82 MgSO4, 88 NaCl, 2.4 NaHCO3, 0.41 CaCl2, 16 Hepes, pH 7.4) supplemented with 100 U.ml^−1^ penicillin, streptomycin and gentamycin.

Channels were characterized in excised inside-out patches at room temperature. Patch pipettes contained (in mM) 154 K^+^, 146 Cl^−^, 5 Mg^2+^, and 10 PIPES-KOH (pH 7.1). They were bathed in solutions which all contained (in mM) 174 K^+^, 40 Cl^−^, 1 EGTA, 1 Mg^2+^, 10 PIPES-KOH (pH 7.1), and methanesulfonate^−^ as the remaining anions. ATP and ADP were added as specified. In those conditions, pipettes had a resistance of ~2 MΩ, except for single-channel recordings where thinner pipettes with a resistance of ~10 MΩ were employed. Membrane potential was −50 mV. Currents were recorded with a Bio-logic RK300 amplifier, filtered at 300 Hz, sampled at 1 kHz, and processed with custom software. Application of the various solutions was performed with a Bio-Logic RSC100 rapid solution changer controlled by custom software. ADP was applied for a time sufficient to reach steady-state, typically 10 s for multichannel recordings and 30–60 s for single-channel recordings. Amplitude histograms were accumulated over 20–40 s before and ~10 s after application of ADP. The tracings shown in the illustrations represent continuous records. Results are displayed as mean ± s.e.m. We excluded from the statistics all patches that showed significant rundown during recording, a very common occurrence when single channels were recorded in the absence of ATP.

Assuming random assembly of WT and mutated SURs, the probability *P*_*n*_ that a tetrameric channel contains n wild-type subunits should follow a binomial distribution:

$$P_{n}={(}_{n}^{4})\cdot p^{n}\cdot {\left(1-p\right)}^{\left(4\;-n\right)}$$

where *p* is the fraction of subunits that are wild-type.

Root-mean-square-deviation (rmsd), an indicator of the distance between experimental data and model predictions, was used as a quantitative measure of the adequacy between the various models and the data.

## Results

**Figure 1:**
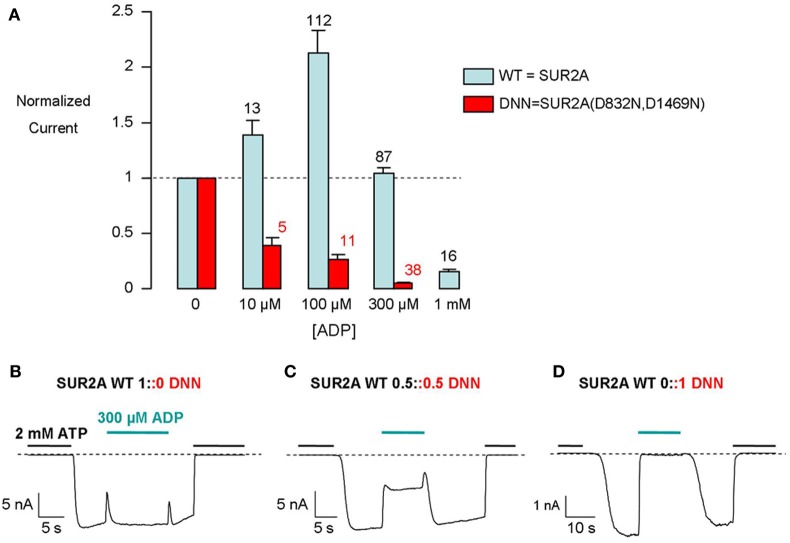
**Distinct responses of WT and mutant K_ATP_ channels to 300 μM ADP as an indicator of SUR-mediated ADP activation. (A)** Concentration-dependent modulation by ADP of WT and mutant K_ATP_ channel currents recorded from inside-out patches. Numbers above bars indicate the number of patches included in the averages. **(B–D)** Representative recordings of macroscopic currents from inside-out patches excised from Xenopus oocytes co-injected with Kir6.2 and the indicated mixtures of WT and mutated (DNN) SUR2A.

Intracellular MgADP has dual opposite effects on K_ATP_ channels. It causes inhibition by binding to the nucleotide site of Kir6.2 and it causes activation by binding to the NBDs of SUR (Dupuis et al., 2008; Proks et al., 2010). Activation requires Mg^2+^ while inhibition does not. In the presence of Mg^2+^, the two effects add up to produce either an increase in channel activity at lower ADP concentrations or a decrease at higher concentrations (Figure 1A).

Activation by MgADP depends on the integrity of the NBDs of SUR and is abolished by mutations of key NBD residues, such as the Walker B aspartates (Gribble et al., 1997b; D'hahan et al., 1999). We mutated these residues in both NBDs (D832N for NBD1 and D1469N for NBD2) of SUR2A. This mutated SUR2A, termed DNN, coexpressed with Kir6.2, formed channels that are no longer activated by ADP (Figure 1A). We interpret these observations by the absence of functional MgADP activatory sites in SUR2A-DNN due to the inability of the mutated NBDs to bind MgADP (Ueda et al., 1997) or to transduce binding into Kir6.2 upregulation.

In 1 mM Mg^2+^, 300 μM ADP elicits little change in the activity of SUR2A+Kir6.2 channels, because activation (through SUR2A) nearly equals inhibition (through Kir6.2) at that concentration (Figure 1B) while it strongly inhibits SUR2A-DNN+Kir6.2 channels (Figure 1D). We selected this concentration in this work as it is a concentration at which inhibition is almost maximal (Figure 1A) but insufficient to mask activation.

How many MgADP-competent SURs are necessary to sustain MgADP activation of the K_ATP_ channel? To estimate this number, we recorded the MgADP response of K_ATP_ channels in Xenopus oocytes that had been injected with various mixtures of the RNAs coding for WT and D832N+D1469N mutant SURs. With an equimolar mixture, channels presented an intermediate sensitivity to MgADP (Figure 1C). Indeed, 300 μM MgADP led to a ~50% decrease in current, midpoint between the effects observed with WT-only and mutant-only channels. Three hypotheses would be consistent with this intermediate sensitivity of the macroscopic currents. Hypothesis 1: WT and mutant subunits cannot coassemble within the same complex: Two populations of channels would exist, ADP-sensitive channels with 4 WT SURs and ADP-insensitive channels with 4 mutant SURs. Hypothesis 2: WT and mutant subunits coassemble randomly and MgADP stimulation varies gradually with mutant subunits content: This could happen if each SUR subunit acted independently to produce a fraction of the maximal activation. Hypothesis 3: WT and mutant subunits coassemble randomly and channels display only 2 phenotypes, WT (full stimulation by MgADP) or mutant (no stimulation), as a function of their subunit composition: This hypothesis would be consistent with one or more SUR subunits triggering a switch of the whole complex from an inactive to a single active state.

**Figure 2:**
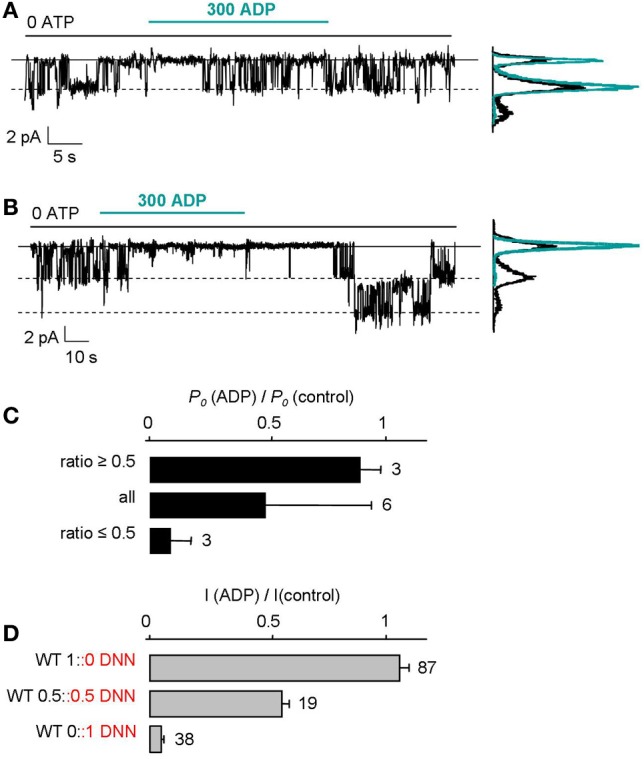
**All-or-none responses of single K_ATP_ channels to MgADP. (A,B)** Recordings of single K_ATP_ channels in inside-out patches excised from oocytes injected with 0.02 ng of Kir6.2 RNA and 0.03 ng each of WT and mutant SUR2A RNAs. Compared to macroscopic current experiments, the amount of RNA injected was reduced 100-fold and smaller patch pipettes were used to lower the channel density toward 1 per patch. Amplitude histograms computed from the current records before (black) and ~10 s after (green) ADP application. **(C)** Using records as in **(A)** and **(B)**, the ratios of the open probabilities (Po) measured after and before ADP application were calculated from amplitude histograms and revealed 2 clusters near 1 (as in **A** where ratio was 0.86) and near 0 (as in **B** where ratio was 0.03). The histogram plots average values of the ratio using either all values, or values above 0.5, or values below 0.5. **(D)** Mean effect of 300 μM ADP on macroscopic currents from WT, mutant, and an equal mix of WT and mutant channels. Numbers beside bars indicate the number of patches included in the averages.

To test hypothesis 2, we examined the effect of ADP on single-channel activity in oocytes expressing an equimolar mixture of WT and mutant subunits (Figure 2). To reduce the number of channels per patch, oocytes were injected with 100-fold less RNA than in the rest of this study and smaller patch pipettes were employed. In spite of these maneuvers, a small fraction of patches contained only 1 or 2 channels which did not fall silent during recording because of rundown. Furthermore, we discarded those patches with 2 channels when only one channel retained robust activity upon ADP application, as this indicated that the 2 channels had probably different number of mutant SUR subunits, one being strongly inhibited by ADP, the other being unaffected. These experiments revealed that channels are either fully inhibited by ADP as in Figure 2B or barely affected as in Figure 2A. The effect of ADP on single channels was quantified by computing NPo from amplitude histograms in control and in 300 μM ADP and by calculating the ratio of the two, yielding Po(ADP)/Po(Control). This value was found to be either close to 1 (ADP-activated) or close to 0 (ADP-inhibited) (Figure 2C). Furthermore, as shown in Figure 2D, the effect of ADP on single ADP-activated channels matched the effect on WT-only macroscopic currents. The same was true for single ADP-inhibited channels and mutant-only macroscopic currents. The data imply a all-or-none effect: Channels are either activated by ADP or insensitive to it. Contrary to hypothesis 2, there is no intermediate ADP activation as a function of the number of mutant subunits.

**Figure 3:**
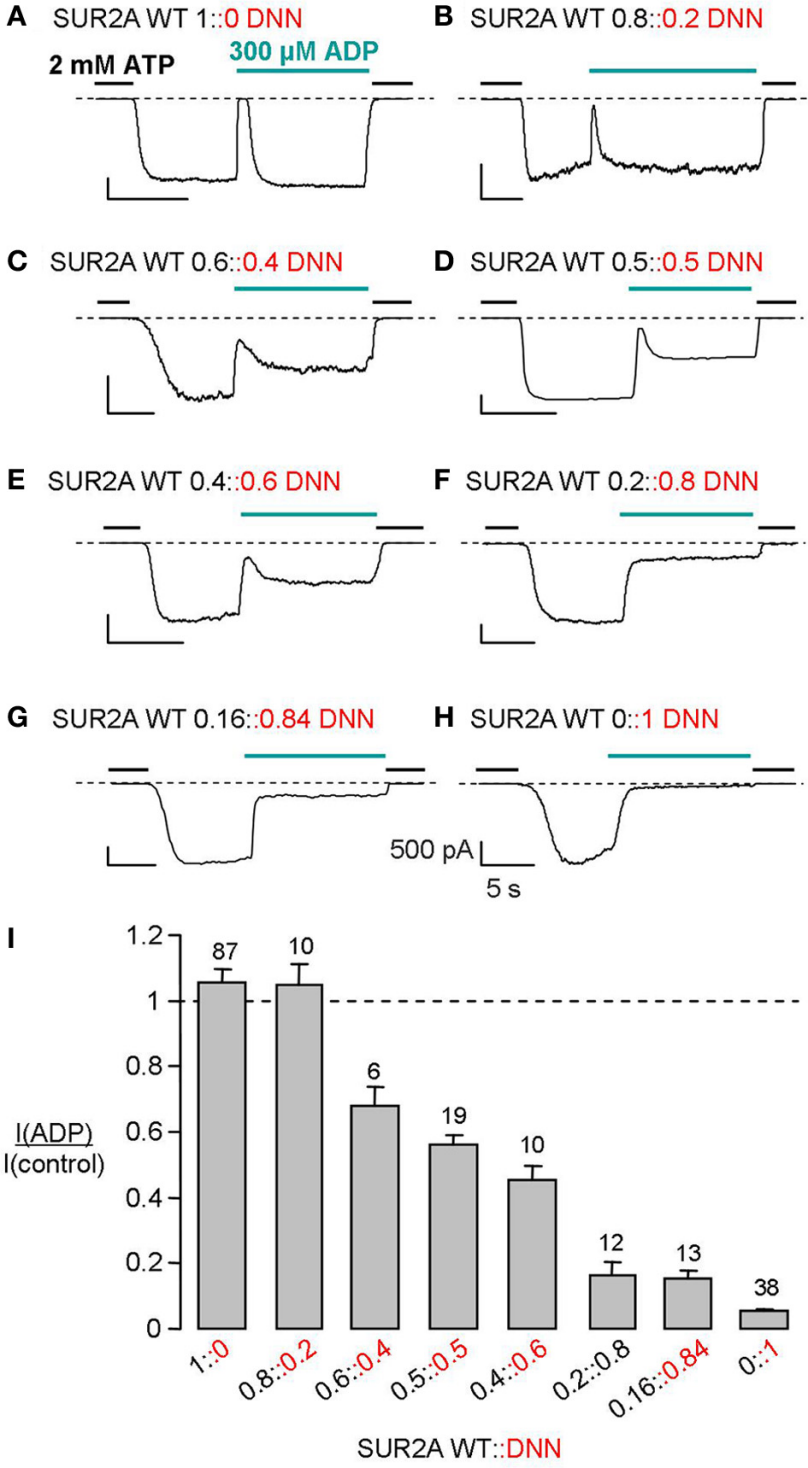
**Effect of ADP varies gradually with the ratio of WT to mutant SUR subunits. (A–H)** MgADP responses of macroscopic currents recorded from Xenopus oocytes co-expressing Kir6.2 and the indicated mixtures of WT and mutant (DNN) SUR2A. **(I)** Average currents recorded in 300 μM ADP normalized to the current measured before in nucleotide-free solution. Numbers above bars indicate the number of patches included in the averages. Normalization was necessary because of the intrinsic variability of the oocyte system. The amplitudes in nA of the currents measured in 0 ATP before ADP application were on average (±s.e.m): 4.4 ± 0.6 (WT 1::0 DNN), 1 ± 0.5 (WT 0.8::0.2 DNN), 1.3 ± 0.4 (WT 0.6::0.4 DNN), 5.3 ± 1.4 (WT 0.5::0.5 DNN), 1.4 ± 0.4 (WT 0.4::0.6 DNN), 1.6 ± 0.7 (WT 0.2::0.8 DNN), 3.5 ± 1 (WT 0.16::0.84 DNN), and 1.5 ± 0.3(WT 0::1 DNN). This variability could not be attributed to a specific construct because experiments performed on the same day with the same batch of oocytes revealed no significant difference.

**Figure 4:**
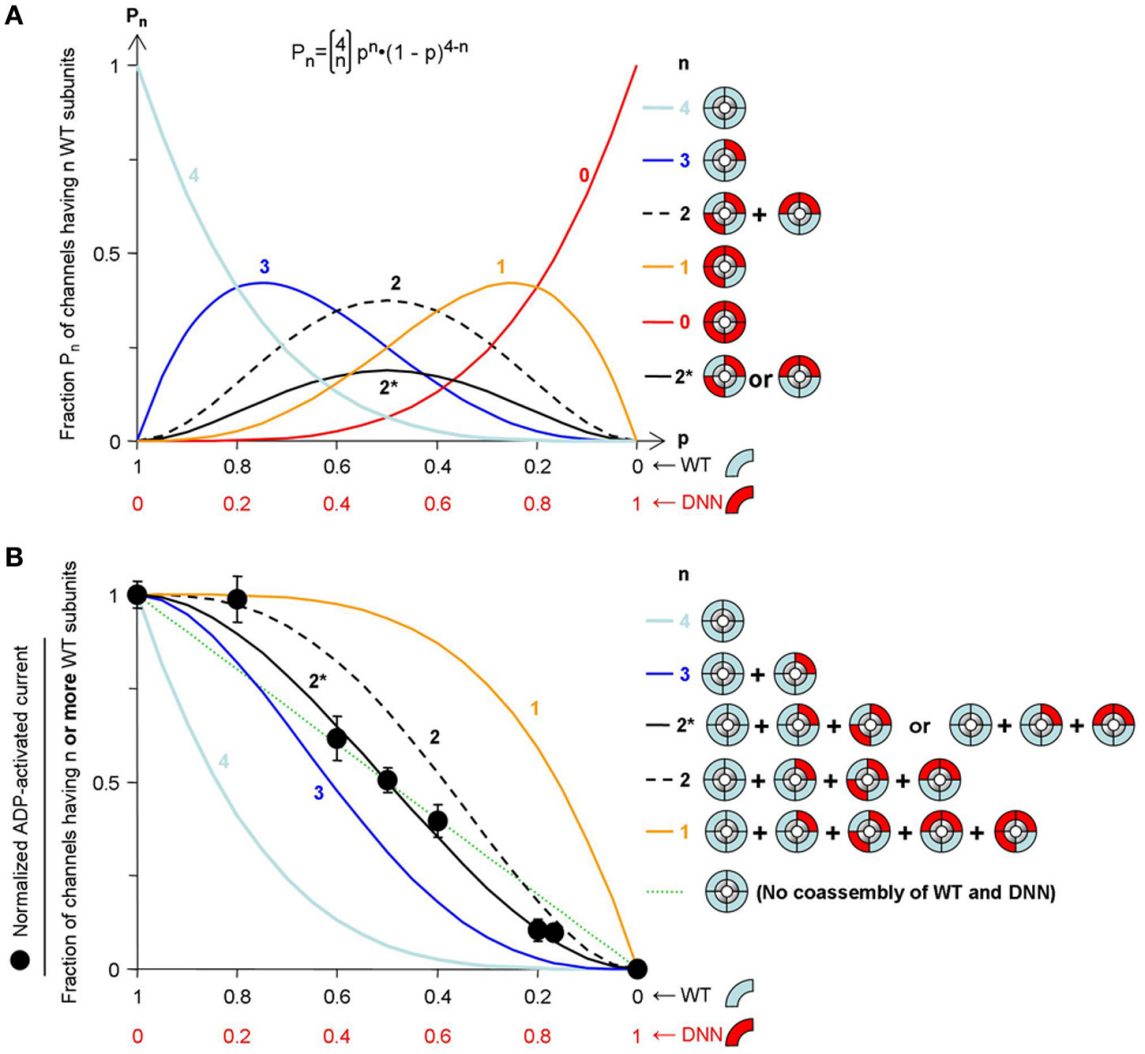
**Model fitting reveals the stoichiometry of ADP activation of the K_ATP_ channel. (A)** Assuming random assembly of WT and mutant subunits, the probability of occurrence of channels having exactly n WT subunits (*P*_*n*_) follows a binomial distribution (equation shown at top). That probability *P*_*n*_ is shown as a function of the fraction of wild-type subunits (p) for each possible value of *n* as indicated above the curves. The label 2^*^ corresponds to the probability of a channel having 2 adjacent (or 2 opposite) WT subunits. **(B)** Probability of a channel having n or more WT subunits calculated using the distributions of **(A)** (n indicated above the curves). The straight dotted line represents the probability of a WT channel if WT and mutant could not co-assemble. The symbols represent normalized ADP-activated current, calculated by normalization of the experimental data of Figure 3I. The rmsd of the experimental data from each model is: 0.327 (*n* = 4), 0.124 (*n* = 3), 0.119 (*n* = 2), 0.352 (*n* = 1), 0.031 (*n* = 2^*^), and 0.071 (no WT/mutant mixing; dotted straight line).

These results are not compatible with hypothesis 2 but do not allow distinguishing hypotheses 1 and 3. Keeping total amount of mRNA constant, we varied the quantities of WT and mutant RNA (WT::DNN). Eight different proportions were co-injected in oocytes. The effects of 300 μM ADP on macroscopic excised-patch currents are illustrated in Figures 3A–H and summarized in the graph of Figure 3I as the ratio of the current in ADP over that in control. This ratio ranged from 0.05 for pure mutant channels to 1.1 for pure WT channels. As a measure of the fraction of channels activatable by ADP for each WT::DNN fraction, we calculated the normalized ADP-activated current as the increment over the pure mutant value (Figure 4B). This value is expected to be proportional to the fraction of ADP-activatable channels if we assume that all WT/mutant combination channels are equally sensitive to inhibition by ADP and that all ADP-activatable combinations are equally sensitive to activation by ADP, as suggested by the single-channel data. Hypothesis 1 (WT and mutant cannot coassemble) predicts that the number of WT, ADP-activated channels should be proportional to the quantity of WT subunits. It corresponds to the straight dotted line in Figure 4B. Experimental data deviate significantly from this line, therefore contradicting hypothesis 1, i.e., WT and mutant subunits do coassemble within the same complex. This conclusion is consistent with reports that SUR1 and SUR2A can randomly assemble in spite of having >500 different residues (Chan et al., 2008; Wheeler et al., 2008). This leaves us with hypothesis 3, random assembly of subunits and two clear-cut, ADP-responsive and ADP-irresponsive, phenotypes. In that case, the fraction of each SUR combination as a function of the relative proportion of mutant subunits should follow a binomial distribution (see Methods) as represented in Figure 4A. Unlike channels possessing 0, 1, 3, and 4 mutant subunits, channels having 2 mutant subunits are of two types: channels with adjacent mutant SUR2A and channels with diametrically-opposite mutant SUR2A. The probability of each type is equal and represented by the solid black line (marked 2^*^) in Figure 4A.

The predictions of Figure 4A yield the fraction of channels having at least n WT subunits, n ranging from 1 to 4, represented as the colored curves in Figure 4B. An additional curve, line 2^*^, is drawn that gives the fraction of channels having at least 2 adjacent WT subunits. The same curve applies to 2 diametrically-opposite WT subunits. Taking rmsd (values in legend of Figure 4) as a measure of the difference between experimental data and model, the experimental data match curve *n* ≥ 2^*^ best with an rmsd >2-fold lower than the linear, no-mixing model and >3-fold lower than any of the other models.

## Discussion

Our results establish that activation of the K_ATP_ channel by MgADP requires interaction with more than 1 SUR subunit and less than 4 subunits, a surprising finding given the expected tetrameric symmetry of the channel. The evidence further suggests interaction with 2 SUR subunits. The position of these subunits appears important. Channels formed by 2 intact SURs and 2 defective SURs can adopt 2 configurations with either adjacent or opposite intact SURs (Figure 4). These configurations are not equivalent with respect to MgADP activation because our experimental data are consistent with one being responsive and the other not. This unusual stoichiometry contrasts with that of other SUR ligands, K_ATP_ channel openers and sulfonylurea blockers, that need interact with only one SUR to affect gating (Dörschner et al., 1999; Gross et al., 1999). Although there is considerable crosstalk in their effects (Gribble et al., 1997a; D'hahan et al., 1999), these modulators target different regions of SUR—MgADP acting on the cytosolic NBDs and openers/blockers acting on the transmembrane domains (Moreau et al., 2000, 2005a; Vila-Carriles et al., 2007). Our conclusion emphasizes a further difference in how these modulators work, suggesting separate mechanisms of action.

It is thought that the K_ATP_ channel complex possess a 4-fold rotational symmetry although the available structural data (Mikhailov et al., 2005; Fotinou et al., 2013) lack sufficient resolution to definitely prove that assertion. A stoichiometry of 2 ADP sites reflects a functional asymmetry that would be consistent with the SURs operating in pairs. Evidence has shown this to be the case for the transporter MRP1, a homologous ABC protein (Yang et al., 2007). One may speculate that SUR modulates gating of Kir6.2 through 2 pathways: a cytoplasmic pathway, used by MgADP, that connects the SUR NBDs to the large cytosolic extension of Kir6.2, and a membrane pathway, used by openers and blockers, that connects the transmembrane domains of the 2 proteins. Indeed, we previously showed that three residues in the cytoplasmic loop connecting TMD2 and NBD2 are essential to mediate ADP activation but not sulfonylurea inhibition (Dupuis et al., 2008). On a molecular model of SUR1 (Bessadok et al., 2011), these residues are on the lateral face of NBD2, well positioned to interact with Kir6.2 and could therefore be part of this cytoplasmic pathway. We have also shown that Kir6.2 gating can be controlled by exerting mechanical force through its cytoplasmic N-terminus (Moreau et al., 2008; Niescierowicz et al., 2014). The functional switch of the cytoplasmic pathway could be a dimer, thus requiring 2 ADP binding events (Ulens and Siegelbaum, 2003), while that of the membrane pathway could be a monomer requiring a single sulfonylurea binding event.

Physiologically, the observed stoichiometry predicts that, in human diseases associated with mutations causing deficient MgADP responses, heterozygous subjects should have half the normal K_ATP_ channel response to ADP. Although free of the severe homozygous symptoms, these individuals might therefore be susceptible to abnormal responses in conditions of metabolic imbalance. This has indeed been reported for mutations of SUR2 that compromise MgADP responsiveness and increase susceptibility to cardiac diseases (Bienengraeber et al., 2004; Olson et al., 2007). In the case of the pancreatic isoform SUR1, mutations that interfere with ADP activation cause mild forms of hyperinsulinism in homozygous carriers (Dunne et al., 2004; Gloyn et al., 2006). Except for rare cases (Huopio et al., 2000; Thornton et al., 2003), heterozygous subjects are not obviously affected although detailed studies on this point are lacking apart from one mutation, V287D (Huopio et al., 2002) that affects channel subunit assembly and trafficking rather than MgADP activation (Chan et al., 2003). Because of the predominant role of K_ATP_ channels in the control of insulin secretion, a 50% impairment should be a cause for concern and could warrant further attention.

### Conflict of interest statement

The authors declare that the research was conducted in the absence of any commercial or financial relationships that could be construed as a potential conflict of interest.
